# Loss of the ciliary protein Chibby1 in mice leads to exocrine pancreatic degeneration and pancreatitis

**DOI:** 10.1038/s41598-021-96597-w

**Published:** 2021-08-26

**Authors:** Benjamin Cyge, Vera Voronina, Mohammed Hoque, Eunice N. Kim, Jason Hall, Jennifer M. Bailey-Lundberg, Gregory J. Pazour, Howard C. Crawford, Randall T. Moon, Feng-Qian Li, Ken-Ichi Takemaru

**Affiliations:** 1 Graduate Program in Molecular and Cellular Pharmacology, Stony Brook University, Stony Brook, NY 11794 USA; 2 Department of Pharmacology, Institute for Stem Cell and Regenerative Medicine, University of Washington School of Medicine and Howard Hughes Medical Institute, Seattle, WA 98195 USA; 3 Graduate Program in Molecular and Cellular Biology, Stony Brook University, Stony Brook, NY 11794 USA; 4 Department of Cancer Biology, Mayo Clinic, Jacksonville, FL 32224 USA; 5 Department of Anesthesiology, McGovern Medical School, The University of Texas Health Science Center at Houston, Houston, TX 77030 USA; 6 Program in Molecular Medicine, University of Massachusetts Medical School, Worcester, MA 01605 USA; 7 Department of Pharmacological Sciences, Stony Brook University, Stony Brook, NY 11974 USA; 8 Department of Pharmacological Sciences, Stony Brook University, BST 7-182, 101 Nicolls Rd., Stony Brook, NY 11794-8651 USA; 9 Present Address: Henry Ford Health System, Detroit, MI 48202 USA

**Keywords:** Ciliogenesis, Mechanisms of disease, Secretion

## Abstract

Primary cilia protrude from the apical surface of many cell types and act as a sensory organelle that regulates diverse biological processes ranging from chemo- and mechanosensation to signaling. Ciliary dysfunction is associated with a wide array of genetic disorders, known as ciliopathies. Polycystic lesions are commonly found in the kidney, liver, and pancreas of ciliopathy patients and mouse models. However, the pathogenesis of the pancreatic phenotype remains poorly understood. Chibby1 (Cby1), a small conserved coiled-coil protein, localizes to the ciliary base and plays a crucial role in ciliogenesis. Here, we report that Cby1-knockout (KO) mice develop severe exocrine pancreatic atrophy with dilated ducts during early postnatal development. A significant reduction in the number and length of cilia was observed in Cby1-KO pancreta. In the adult Cby1-KO pancreas, inflammatory cell infiltration and fibrosis were noticeable. Intriguingly, Cby1-KO acinar cells showed an accumulation of zymogen granules (ZGs) with altered polarity. Moreover, isolated acini from Cby1-KO pancreas exhibited defective ZG secretion in vitro. Collectively, our results suggest that, upon loss of Cby1, concomitant with ciliary defects, acinar cells accumulate ZGs due to defective exocytosis, leading to cell death and progressive exocrine pancreatic degeneration after birth.

## Introduction

Cilia are evolutionarily conserved microtubule-based organelles that protrude from the apical cell surface and perform diverse biological functions^1–3^. Primary cilia are comprised of a 9 + 0 microtubule arrangement, present on a wide range of cell types, and play crucial roles in mechanosensation and intracellular signaling. On the other hand, motile multicilia are comprised of a 9 + 2 structure and found on epithelial cells lining the respiratory tract, oviduct, and brain ventricles. They are important for clearing airway mucus and debris, transporting ova from the ovary to the uterus, and circulating cerebrospinal fluid in the brain. Cilia are typically assembled from the basal body, which is derived from the mother centriole. The mother centrioles harbor accessory structures, including subdistal and distal appendages. The distal appendages (also called “transition fibers” at the ciliary base) are critical for the recruitment of small vesicles and subsequent docking of basal bodies to the plasma membrane^4–7^. Since no protein synthesis occurs in cilia, ciliary proteins are transported from the cell body via polarized vesicle trafficking^8,9^. The extension of a cilium and its maintenance requires intraflagellar transport (IFT), a bidirectional transport system that tracks along the axonemal microtubules^10^.

Genetic defects in the structure and function of cilia are associated with pleiotropic disorders termed ciliopathies^1–3^. Dysfunctional primary cilia are linked to various diseases such as polycystic kidney disease (PKD) and Bardet-Biedl syndrome (BBS). Their clinical features are variable but include *situs inversus*, retinal degeneration, intellectual disability, and cystic kidney, liver, and pancreas. On the other hand, defective multicilia are prominently associated with primary ciliary dyskinesia (PCD). PCD patients manifest chronic respiratory infections, infertility, and hydrocephalus^11–13^.

Chibby1 (Cby1) is a 15-kDa coiled-coil protein that is evolutionarily conserved in animals with motile cilia^14,15^. Cby1 localizes to the distal appendages and transition fibers and plays a critical role in formation of both primary cilia and multicilia^5,15–22^. We demonstrated that CEP164 (also known as NPHP15), which is mutated in human ciliopathies including nephronophthisis and BBS^23,24^, directly interacts with and recruits Cby1 to the distal appendages during ciliogenesis^5^. Cby1 then interacts with the membrane trafficking machinery component Rabin8, a guanine nucleotide exchange factor (GEF) for the small GTPase Rab8, and recruits Rab8 to facilitate the assembly of ciliary membranes at centrioles and basal bodies. More recently, we identified the Cby1 interactors, the membrane-binding Bin/Amphiphysin/Rvs (BAR)-domain containing proteins, ciBAR1 (Cby1-interacting BAR domain-containing 1) and ciBAR2 (formerly known as FAM92A and FAM92B)^25^. ciBAR1 and ciBAR2 are recruited to mother centrioles and basal bodies by Cby1 to facilitate ciliogenesis likely through regulation of membrane remodeling processes. Cby1 also binds to the ciliary proteins Dzip1 and Dzip1-like (Dzip1L) during ciliogenesis^26,27^. Recently, it was reported that loss of Cby1 causes a ciliopathy with features of Joubert syndrome^28^.

Germline Cby1-knockout (KO) mice display several hallmarks of ciliary defects, including chronic upper airway infection^16^, polycystic kidneys^19^, and reduced fertility as well as hydrocephalus and polydactyly at low frequency. In *D. melanogaster*, Cby1 is expressed in sensory neurons and male germ cells, the only ciliated cell types in this organism, and is required for proper formation of neuronal cilia and sperm flagella^15,22^. Similarly, in *X. laevis*, Cby1 is indispensable for the ciliogenesis of multiciliated cells in the epidermis^20^. These studies highlight an evolutionarily conserved, critical function for Cby1 in ciliogenesis. Cby1 is the ubiquitous, most prominent family member. There are two other Cby family members Cby2 (also known as Nurit or Spert)^29^ and Cby3 in mammals, but their functions are unknown.

The mammalian pancreas consists of roughly 95% exocrine tissue that secretes digestive enzymes and 1–3% endocrine tissue that produces hormones such as insulin. In the adult pancreas, primary cilia have been found on ductal and centroacinar/terminal ductal cells in the exocrine region as well as on endocrine α-, β-, and δ-cells, but exocrine acinar cells lack primary cilia^30–34^. Pancreatic lesions including atrophy, cysts, fibrosis, and pancreatitis have been reported in ciliopathy patients^35–37^ as well as mouse models^30–32,36,38^. In ciliopathy mouse models mutant for IFT88 (also known as polaris)^30,32^ or KIF3A^31^, primary cilium dysfunction in the pancreas leads to ductal hyperplasia in parallel with massive apoptosis of neighboring acinar cells. Interestingly, in these mouse models, acini are severely affected, whereas endocrine cell differentiation and architecture appear relatively normal. The molecular and cellular bases underlying their pancreatic pathologies remain poorly understood. However, these studies proposed a model in which defective primary cilia in the ductal epithelium causes impaired sensing of luminal flow and obstruction of pancreatic ducts. This results in the aberrant release of digestive enzymes into the tissue parenchyma and subsequent destruction of surrounding acinar cells.

Here, we report that Cby1-KO mice show a rapid, progressive degeneration of pancreatic acinar cells after birth. In agreement with this, the number and length of primary cilia was significantly reduced in Cby1-KO pancreas compared to wild-type (WT) pancreas. Intriguingly, Cby1-KO acinar cells showed an accumulation of zymogen granules (ZGs) that were mis-polarized and dispersed throughout the cytoplasm as early as at postnatal day (P) 0. Live-cell imaging using the lipid fluorescent probe FM1-43 indicated that acini isolated from Cby1-KO pancreas exhibit defective ZG secretion. Taken together, our results suggest that Cby1 plays a crucial role in ciliogenesis in the pancreas and that Cby1-KO acinar cells accumulate ZGs throughout the cytoplasm due to defective exocytosis, leading to cell death and rapid exocrine pancreatic degeneration.

## Results

### Progressive exocrine pancreatic degeneration in Cby1-KO mice

**Figure 1:**
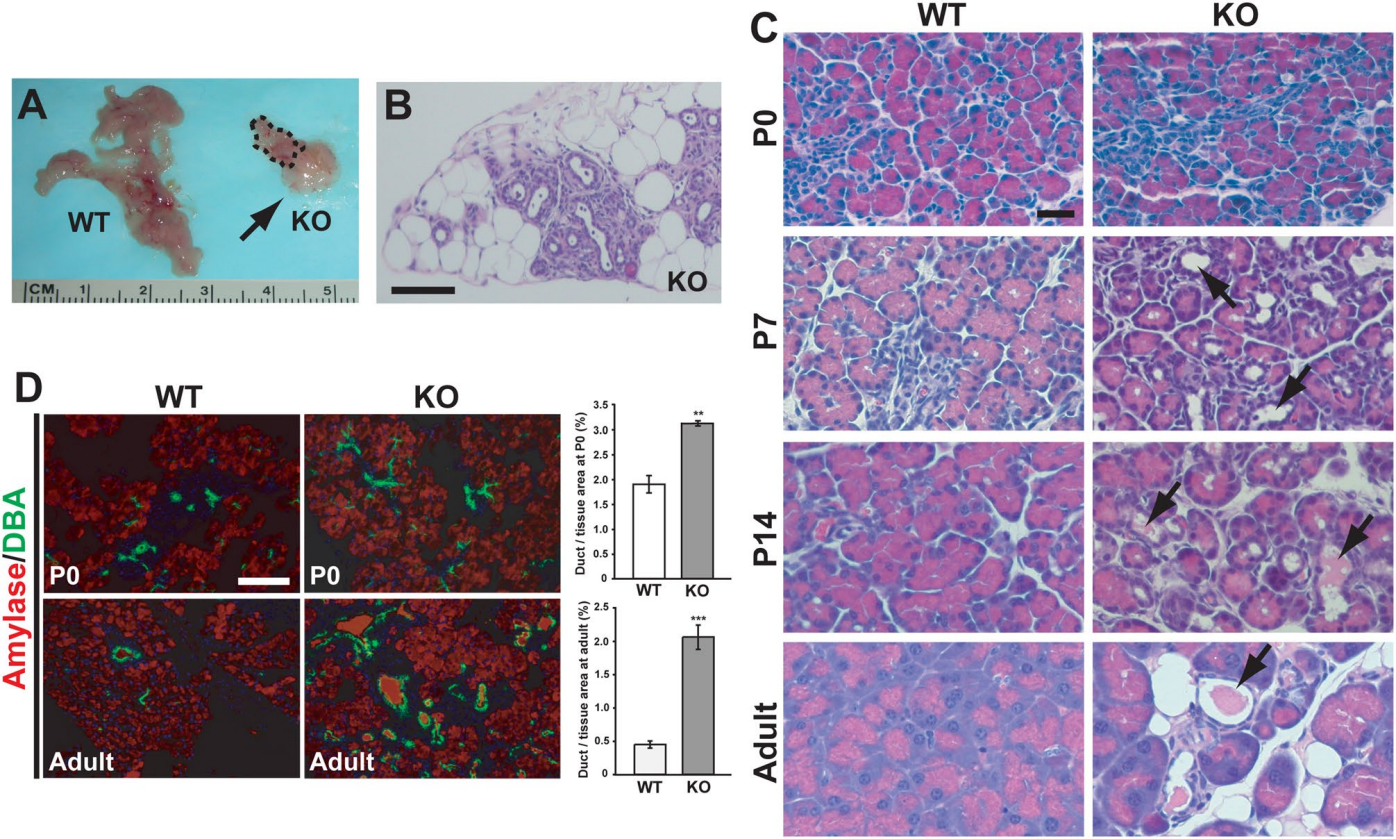
Progressive exocrine pancreatic degeneration in Cby1-KO mice after birth. (**A**) Gross morphology of adult pancreata. The Cby1-KO pancreatic tissue is encircled by a dotted line. The arrow denotes ectopic adipose mass in the Cby1-KO pancreas. (**B**) H&E staining of the adult Cby1-KO pancreas with extensive lipomatosis. Scale bar, 200 μm. (**C**) Pancreatic sections of WT and Cby1-KO mice at the indicated ages were stained with H&E. Arrows indicate dilated ducts. Scale bar, 20 μm. (**D**) Ductal expansion in the pancreas of Cby1-KO mice. Pancreatic sections of P0 and adult mice were stained with DBA-lectin (ductal cell marker, green) and α-amylase antibody (acinar cell marker, red). Mean ratios of ductal to total tissue areas ± SEM are shown on the right. For each group, three individual animals were used for IF staining, and five non-overlapping fields per animal were photographed for quantification. Scale bar, 50 μm. **p < 0.01; ***p < 0.001.

Through gross necropsy of various organs and tissues, we found that Cby1-KO mice consistently exhibit pancreatic atrophy with massive ectopic fat depots (Fig. 1A,B). The pancreas-to-body weight ratio of adult Cby1-KO mice was 29.6% of that of WT mice at 2 months of age: 0.00887 ± 0.00025 (SEM) for WT vs. 0.00263 ± 0.0001 for KO (n = 4). Upon histological examination, their pancreas appeared normal at birth (Fig. 1C, P0), but quantitative analysis of DBA lectin-positive ductal vs. amylase-positive exocrine tissue areas revealed a 1.7-fold increase in ductal areas in Cby-KO mice (Fig. 1D). Within 1–2 weeks, Cby1-KO pancreas manifested severe acinar cell loss with the appearance of enlarged ducts and mucus accumulation (Fig. 1C, P7 and P14, arrows). Necrotic acinar cells were noticeable as early as at P3 (data not shown). Pancreatic abnormalities in adult Cby1-KO mice included disorganized acinar morphology, profound ductal dilation, mucus accumulation, and lipomatosis (Fig. 1C, Adult). Ductal expansion progressed dramatically in adult Cby1-KO mice with a greater than fourfold increase in ductal areas compared to WT controls (Fig. 1D).

**Figure 2:**
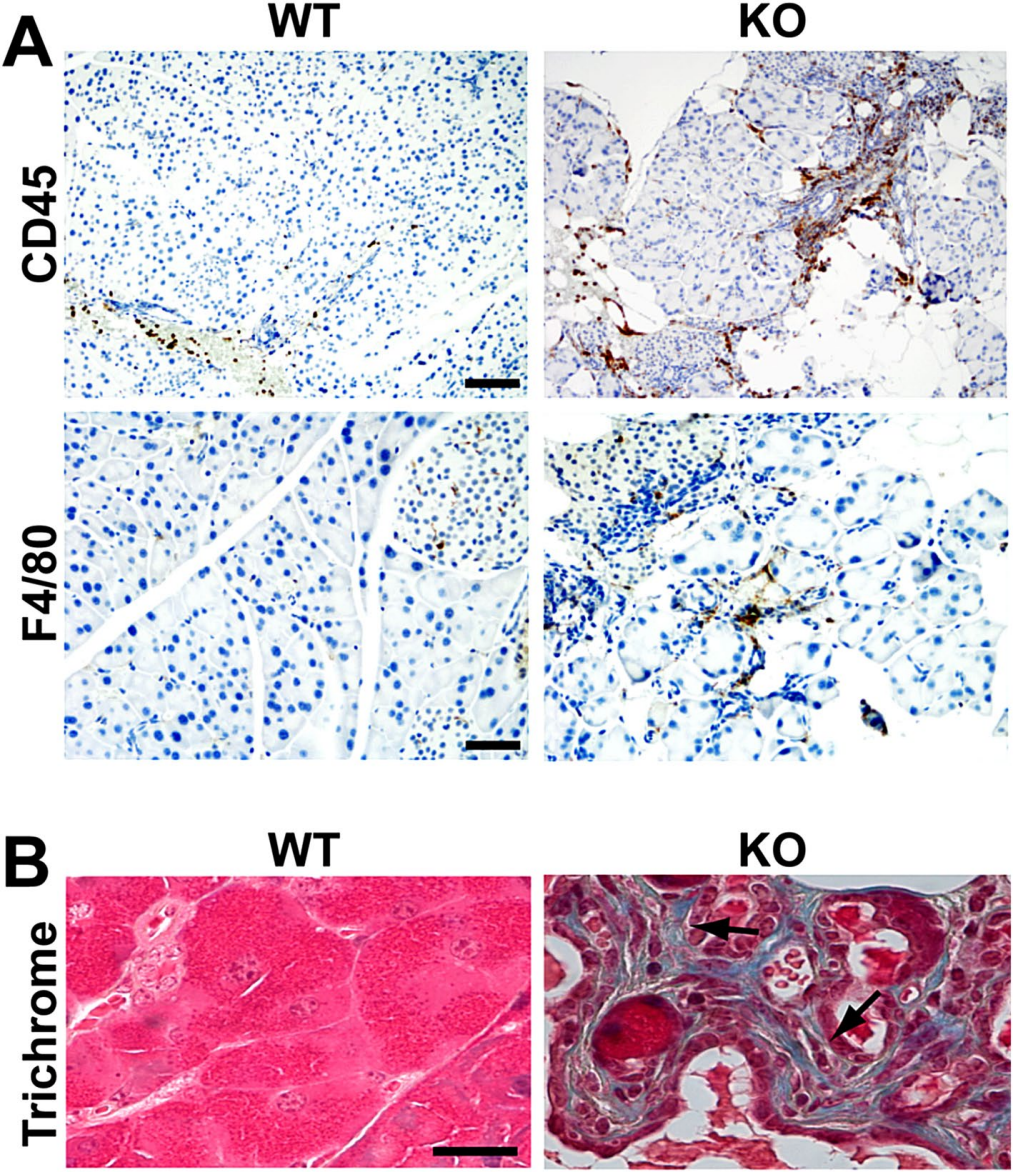
Fibrosis and chronic inflammation in the Cby1-KO pancreas. (**A**) Pancreatic sections from adult mice were immunostained with antibodies for the inflammation markers CD45 (leukocytes) and F4/80 (macrophages). Scale bars: CD45, 100 μm; F4/80, 50 μm. (**B**) Pancreatic sections from adult mice were stained with Trichrome to detect collagen deposition. Blue staining indicates fibrosis (arrows). Scale bar, 20 μm.

Chronic pancreatitis is a progressive inflammatory disease characterized by inflammation, fibrosis, and acinar cell atrophy, leading to irreversible damages over time^39,40^. To examine if Cby1-KO mice show any signs of chronic pancreatitis, we performed immunohistochemistry for inflammatory markers. As shown in Fig. 2A, there was a pronounced increase in the number of CD45-positive leukocytes and F4/80-positive macrophages in adult Cby1-KO pancreatic tissues. Furthermore, trichrome staining to detect collagen deposition revealed extensive fibrosis in the Cby1-KO pancreas (Fig. 2B, arrows), consistent with chronic pancreatitis.

**Figure 3:**
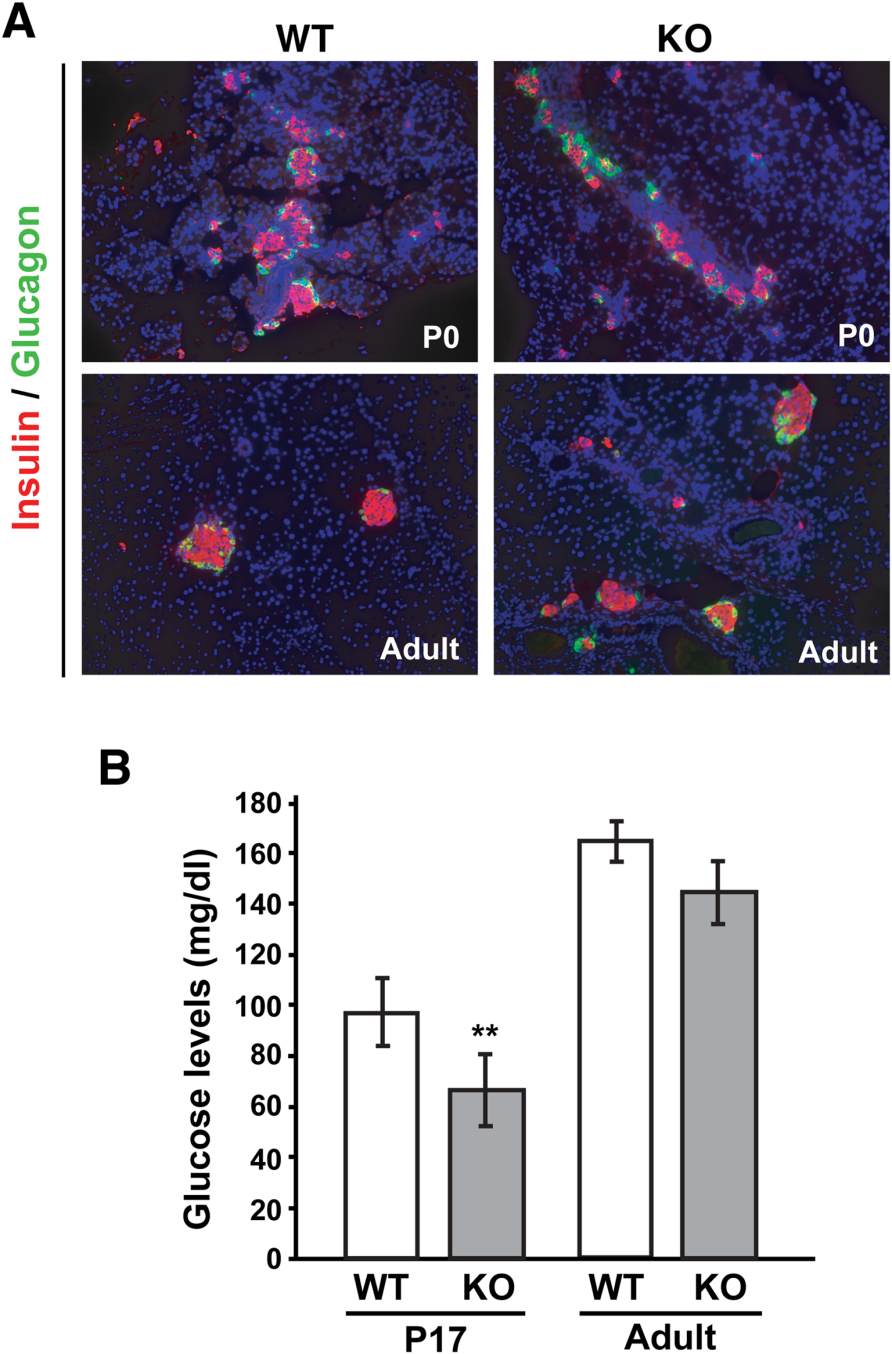
Characterization of the endocrine pancreas. (**A**) Pancreatic sections of P0 and adult mice were immunostained for the β-cell marker insulin and α-cell marker glucagon. (**B**) Blood glucose levels were measured in P17 and adult mice. Data represent means ± SEM. **p < 0.01.

In contrast, endocrine islets were not overtly affected in the pancreas of both P0 and adult Cby1-KO mice with normal endocrine cell differentiation and architecture as examined by immunofluorescence (IF) staining for insulin (β-cell marker) and glucagon (α-cell marker) (Fig. 3A). We also measured blood glucose levels in P17 and adult Cby1-KO and WT mice. Cby1-KO mice exhibited lower blood glucose levels at P17, likely caused by malnutrition since Cby1-KO pups display growth retardation in early postnatal days^16^. In adult mice, however, there was no statistically significant difference in glucose levels between the two genotypes (Fig. 3B). Taken together, our data suggest that ablation of Cby1 results in progressive loss of exocrine acinar cells concomitant with chronic pancreatitis, while overall endocrine architecture and function appear normal.

### Proliferation and apoptosis in the pancreas of Cby1-KO mice

**Figure 4:**
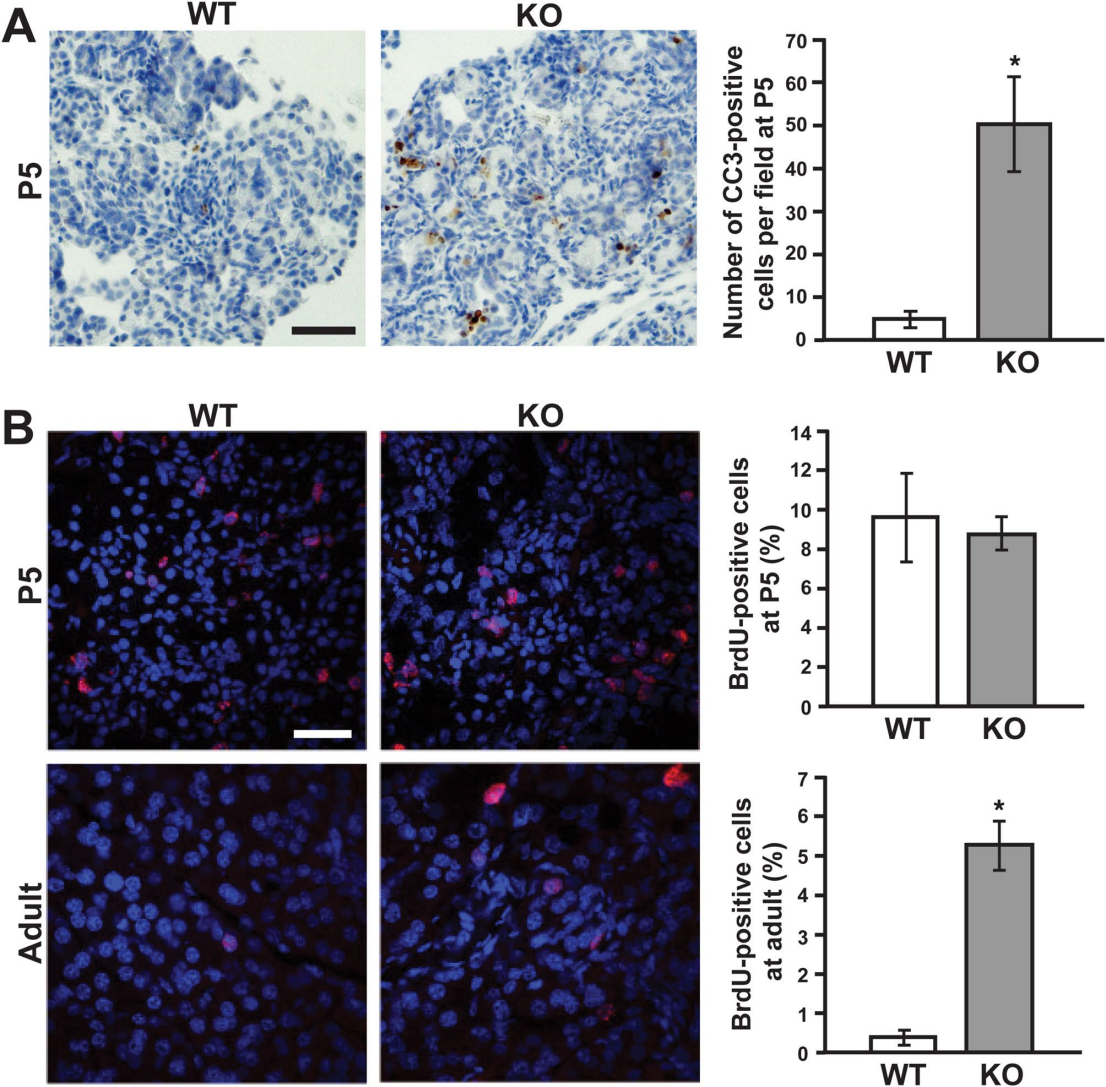
Apoptosis and proliferation in the pancreas. (**A**) Pancreatic sections from P5 mice were immunostained with cleaved caspase-3 (CC3) antibody and counterstained with hematoxylin to assess the level of apoptosis. Quantification represents the average of 15–20 fields (20 × objective) (n = 3 per genotype). Data represent means ± SEM. Scale bar, 50 μm. *p < 0.05. (**B**) Pancreatic sections from P5 and adult mice that were injected with BrdU were labeled with anti-BrdU antibody (red) to examine the level of proliferation. Nuclei were visualized with DAPI. The number of cells was counted in at least 10 random 63 × objective fields (n = 3 per genotype), and the percentage of BrdU-positive cells was calculated. Data represent means ± SEM. *p < 0.05. Scale bar, 20 μm.

To gain insight into the basis of the pancreatic atrophy in Cby1-KO mice, we examined proliferation and apoptosis using BrdU incorporation assays and cleaved caspase-3 (CC3) staining, respectively. As expected, the number of apoptotic cells was dramatically elevated tenfold in the Cby1-KO pancreas compared to the WT pancreas at P5 (Fig. 4A). A similar trend was observed using TUNEL assays (Supplementary Fig. 1A) Interestingly, there was a 16-fold increase in proliferation in the adult Cby1-KO pancreas compared to the WT pancreas, whereas no significant changes were detected at P5 (Fig. 4B). Increased proliferation was also detected using IF staining for phospho-histone H3 (Ser10) in adult Cby1-KO pancreas (Supplementary Fig. 1B). These results suggest that the Cby1-KO pancreas elicits a compensatory proliferative response after damage, but acinar cells fail to survive, leading to loss of the exocrine tissue.

### Localization of Cby1 in the pancreas

**Figure 5:**
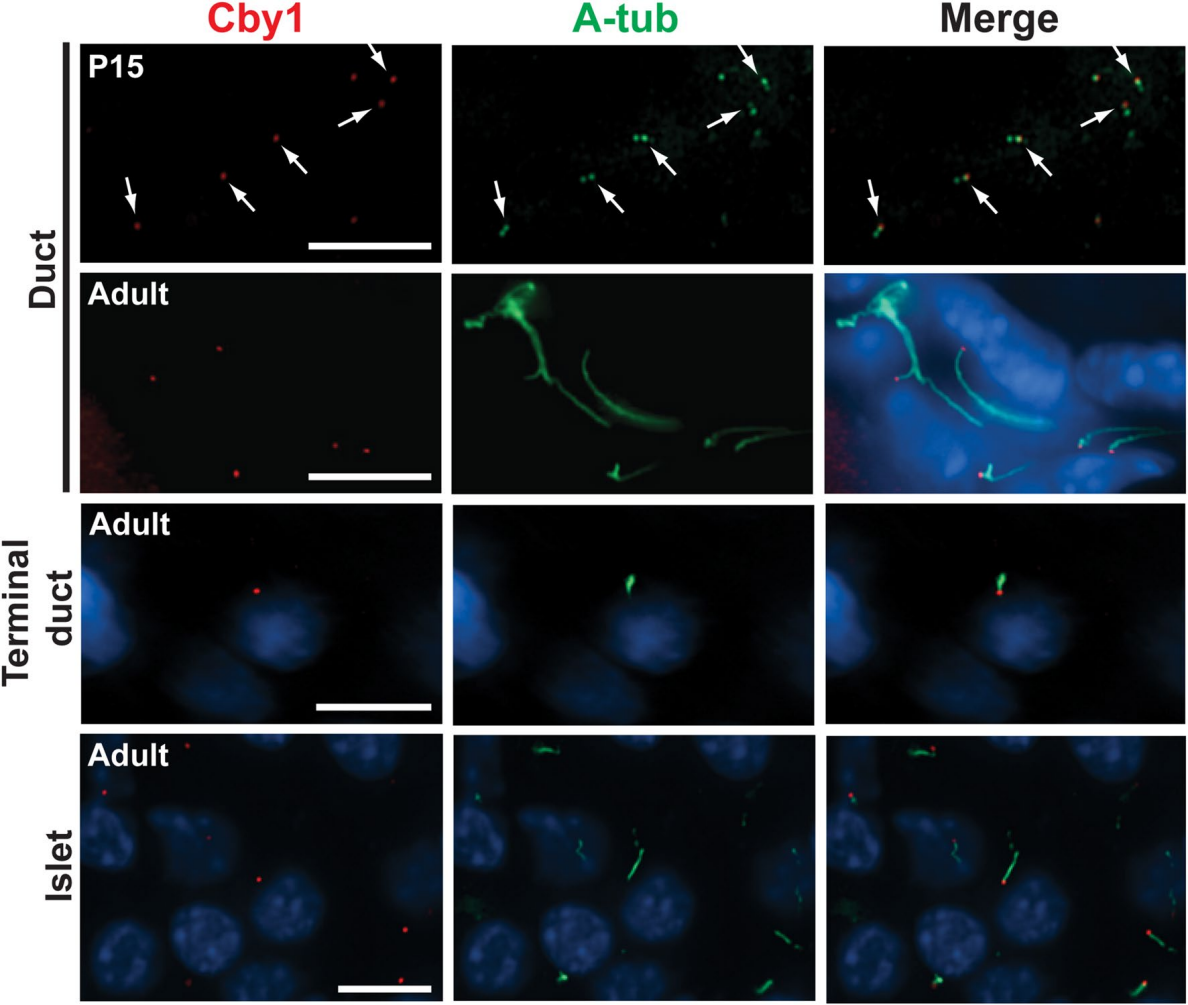
Localization of Cby1 in the pancreas. Pancreatic sections from P15 and adult mice were immunostained with antibodies against Cby1 (red) and acetylated α-tubulin (A-tub) (green). Nuclei were detected with DAPI (blue). In undifferentiated ductal cells at P15, which had not yet elongated primary cilia, Cby1 localized to only one of the two centrioles (arrows). In the adult pancreas, intense Cby1 signals were seen at the base of primary cilia in ductal, terminal ductal, and islet β-cells. Scale bar, 10 μm.

We previously demonstrated that Cby1 localizes to the base of cilia and plays a critical role in ciliogenesis^5,17–19^. In the pancreas, primary cilia are present on the apical surface of ductal, terminal ductal/centroacinar, and islet cells but not acinar cells^30,32,34,41^. To determine the localization of Cby1 in the pancreas, we performed IF staining for the centriolar/ciliary marker acetylated α-tubulin (A-tub). In developing ducts at P15, Cby1 was detected at one of the two A-tub-positive centrioles in each non-ciliated cell (Fig. 5, arrows). It is most likely the mother centriole since Cby1 is predominantly found there in other cell types^18,19^. In the adult pancreas, Cby1 was clearly detectable at the base of primary cilia in ductal, terminal ductal/centroacinar, and islet cells (Fig. 5). Using the indirect IF technique, we were not able to reliably detect Cby1 protein in acinar cells. However, our RT-PCR data indicate that Cby1 is expressed in acinar cells isolated by FACS (Supplementary Fig. 2A). In addition, a single-cell RNA sequencing analysis suggests that acinar cells are heterogeneous, and Cby1 is expressed in a subpopulation of acinar cells (Supplementary Fig. 2B)^42^. The localization of Cby1 at centrioles and basal bodies suggests that Cby1 plays a role in ciliogenesis in the pancreas.

### Defective primary cilia in the Cby-KO pancreas

**Figure 6:**
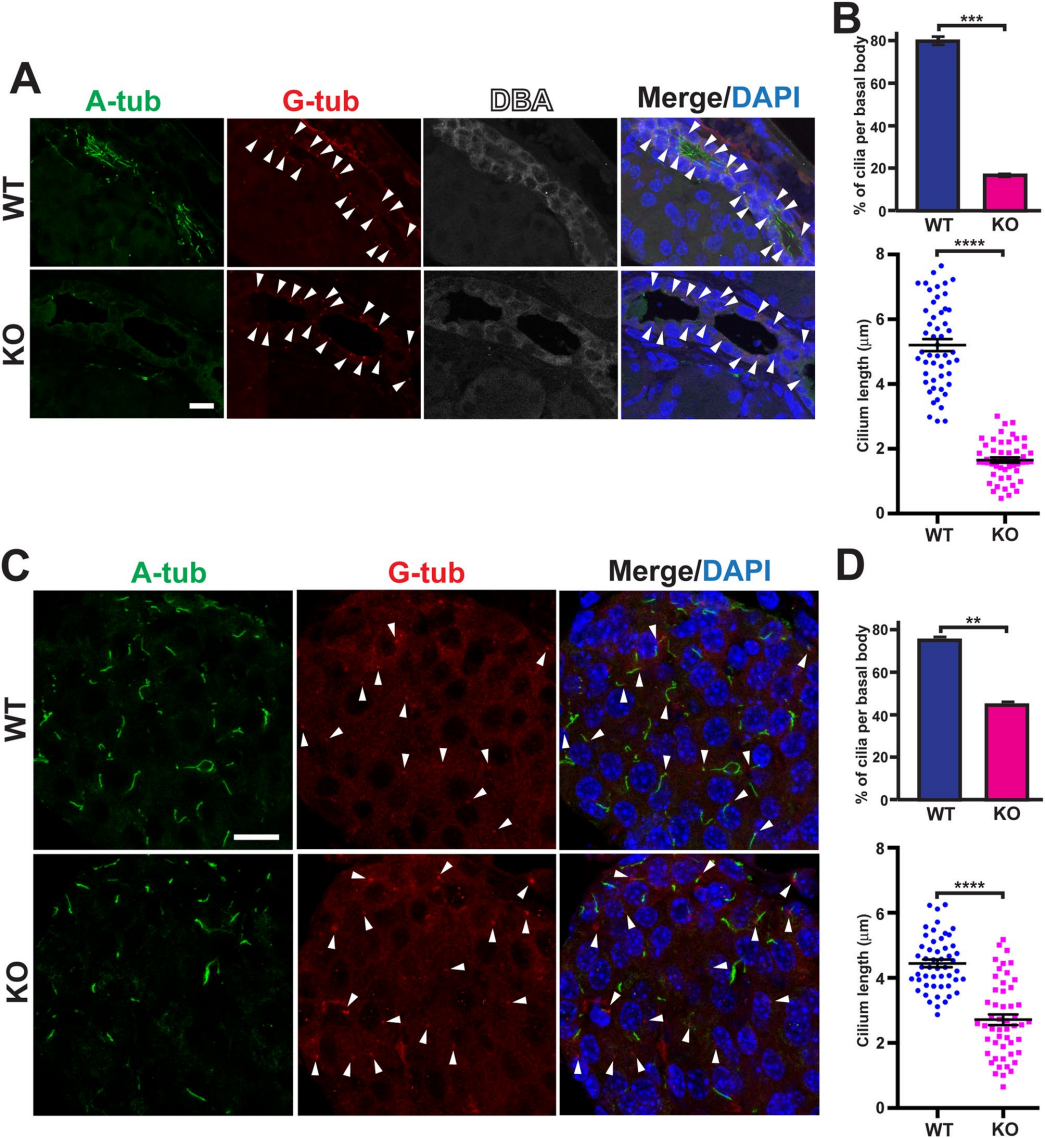
Cby1-KO pancreata show a significant decrease in the number and length of primary cilia. (**A**, **C**) Pancreatic sections from P18 mice were labeled for A-tub (green), γ-tubulin (G-tub, red), and DBA (white) to evaluate the status of primary cilia in ducts (**A**) and islets (**C**). Nuclei were visualized with DAPI. Arrowheads denote basal bodies. Scale bars, 10 μm. (**B**, **D**) The number and length of primary cilia were quantified based on results represented in (**A**, **C**). For the percentage of cilia per basal bodies, data are the average of three independent experiments with n = 70 per category per experiment for each genotype. For quantification of cilium lengths, a total of 51 cilia were quantified per category for each genotype. Data represent means ± SEM. **p < 0.01, ***p < 0.001, ****p < 0.0001.

To investigate whether primary cilia are perturbed in the Cby-KO pancreas, we performed IF staining for A-tub and the basal body marker γ-tubulin (G-tub) at P18 (Fig. 6). There was a 76% decrease in the number of primary cilia in Cby1-KO ducts, as revealed by DBA lectin costaining, compared to WT ducts (Fig. 6A,B). In addition, the length of ductal cilia was dramatically reduced in the Cby1-KO pancreas (1.7 ± 0.08 μm Cby1-KO cilia vs. 5.2 ± 0.19 μm WT cilia) (Fig. 6B). Similarly, there was a 41% decrease in the number of primary cilia in Cby1-KO islets compared to WT islets (Fig. 6C), and the length of islet cilia was also reduced at P18 (2.7 ± 0.16 μm Cby1-KO cilia vs. 4.4 ± 0.12 μm WT cilia) (Fig. 6D). We noticed that some ductal primary cilia appeared highly elongated, reaching about 8 μm. Profound ciliary defects in the Cby1-KO pancreas persisted into adulthood as revealed by IF staining for the ciliary membrane marker Arl13b and A-tub (Supplementary Fig. 3). Arl13b showed extensive overlap with A-tub in both ducts and islets of the Cby1-KO pancreas, indicating no aberrant ciliary membrane assembly (Supplementary Fig. 3).

**Figure 7:**
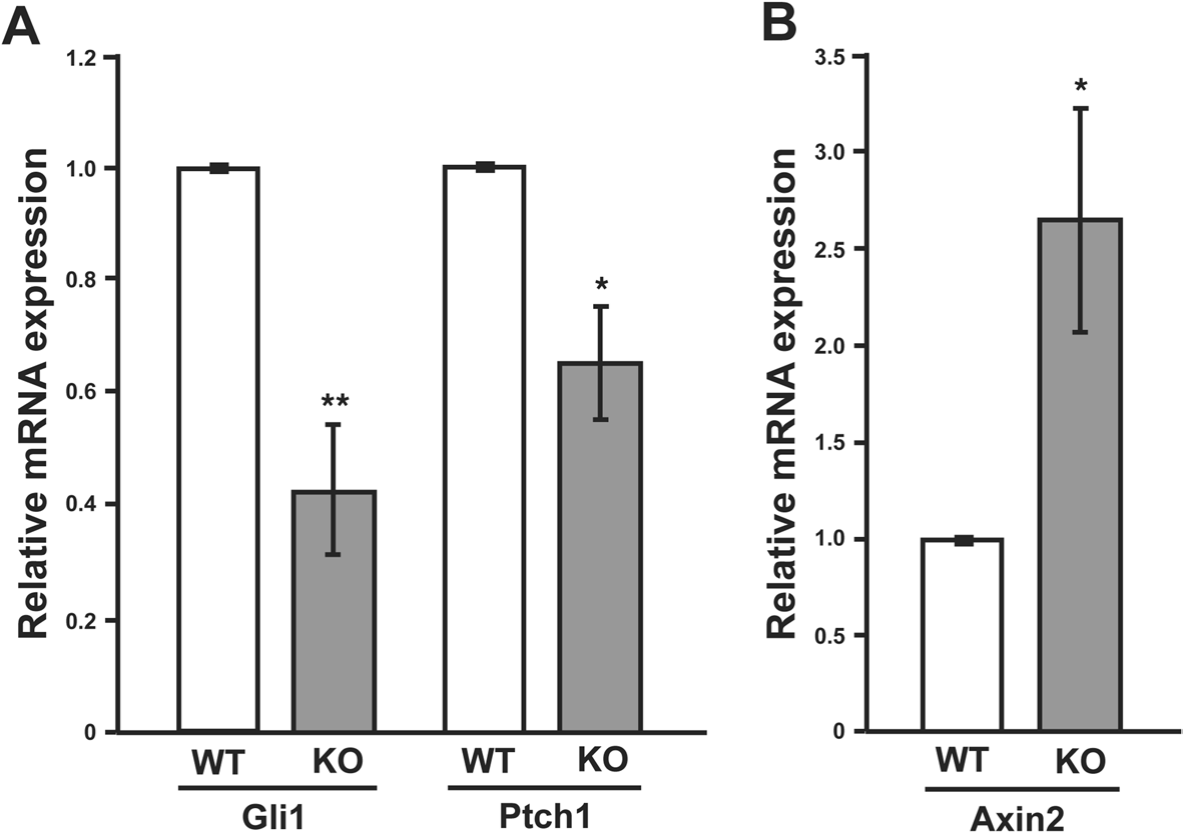
Altered ciliary signaling in the Cby1-KO pancreas. Real-time PCR analysis was performed for expression levels of the direct Hh target genes Gli1 and Ptch1 (**A**) and canonical Wnt target gene Axin 2 (**B**) in the adult pancreas (n = 3 per genotype). WT values were set as 1. Data represent means ± SEM. *p < 0.05; **p < 0.01.

Primary cilia are essential for the transduction of Hedgehog (Hh) signaling in mammals^2,12^. In agreement with this, the expression of the Hh target genes such as Gli1 and Patched1 (Ptch1) was diminished in the Cby1-KO pancreas (Fig. 7A). In addition, primary cilia have been reported to negatively influence canonical Wnt/β-catenin signaling^43,44^. Consistent with this, the expression of the direct β-catenin target Axin 2 was elevated in the Cby1-KO pancreas (Fig. 7B). These data underscore the importance of Cby1 function in ciliogenesis in the pancreas.

### Altered polarity and defective secretion of zymogen granules in the Cby1-KO pancreas

**Figure 8:**
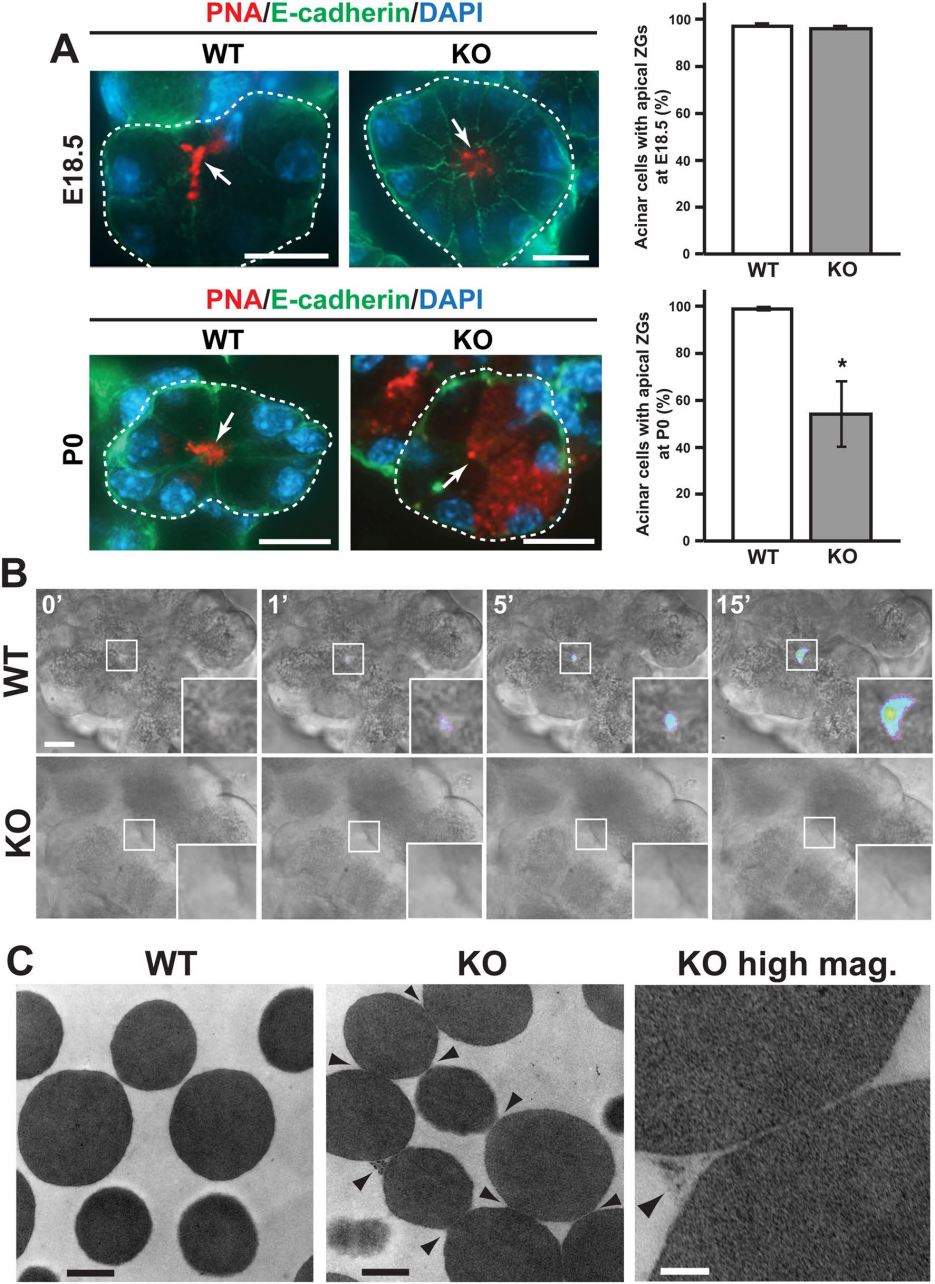
Defective ZG polarity and exocytosis in Cby1-KO acinar cells. (**A**) Pancreatic sections from E18.5 and P0 mice were labeled with PNA-lectin (ZG marker, red) and E-cadherin antibody (basolateral membrane marker, green) as indicated. Nuclei were visualized by DAPI. Acini are encircled with dotted lines. The apical lumen is indicated by arrows. The quantification of acinar cells with apical ZGs is shown on the right. More than 200 cells were counted per age for each genotype. Data represent means ± SEM. *p < 0.05. (**B**) Acini were isolated from the pancreas of adult mice, incubated with FM1-43, and stimulated with cerulean at time 0. Images were taken 1 frame/min for ~ 30 min. Representative sequence images in pseudocolor are shown to better visualize the fluorescent intensities. The squared areas with the apical pole of an isolated acinus are enlarged in the insets. Scale bars, 10 μm. (**C**) ZGs purified from adult pancreata were subjected to TEM. Note that ZGs from Cby1-KO acinar cells were interlinked with each other by a proteinaceous material (arrowheads). Scale bars: 500 nm; KO high mag., 100 nm.

In an effort to understand the mechanistic basis of the exocrine pancreatic insufficiency in Cby1-KO mice, we went on to investigate early changes in acinar cells by visualizing zymogen granule (ZG) distribution using the lectin peanut agglutinin (PNA), which has been shown to detect apical zymogen granules^45,46^. At embryonic day (E) 18.5, ZGs were correctly localized at the apical cell surface in Cby1-KO acinar cells (Fig. 8A). However, significant mis-polarization of ZGs was noticeable in Cby1-KO acini as early as at P0 even before any histological abnormalities were evident (Fig. 8A). On average, 55% of Cby1-KO acinar cells exhibited altered ZG localization. Overall, the apical-basal polarity of the acinar cells appeared normal as nuclei were correctly positioned in the basal region. This suggests that Cby1-KO acinar cells mature normally during embryonic development but manifest mis-polarization of ZGs postnatally once the pancreas begins to function after feeding, leading to cell death.

Pancreatic acinar cells are responsible for the production and secretion of various digestive enzymes, including amylases and lipases, to aid food digestion in the small intestine. To meet the high daily demand for these enzymes, acinar cells exhibit one of the highest rates of protein synthesis and secretion among all mammalian cell types^47^. Pancreatic acinar cells serve as an excellent model system to study vesicle trafficking and polarized secretion as massive exo- and endocytic events can be triggered in response to a stimulus. To directly assess a possible defect in the exocytosis of ZGs in Cby-KO acinar cells, we performed live imaging of isolated acini exposed to the membrane fluorescent dye FM1-43 in vitro as described previously^48–50^. Stimulation of ZG secretion with cerulean (a cholecystokinin analog) evoked a noticeable increase in FM1-43 fluorescence at the apical lumen of Cby-WT acini, but no such hot spots were apparent in Cby1-KO acini (Fig. 8B and Supplementary Videos 1 and 2). These data imply that Cby1-KO acinar cells succumb to cell death due to defective secretion and resultant intracellular accumulation of ZGs.

### Interlinking of ZGs in Cby1-KO acinar cells

To gain further insight into the abnormal ZG polarity and defective secretion in Cby1-KO acinar cells, we examined the morphology of ZGs at the ultrastructural level. ZGs were purified from pancreatic acinar cells of adult Cby1-WT and KO mice and processed for transmission electron microscopy (TEM) (Fig. 8C). While WT ZGs showed a typical appearance of large and electron-dense granules that were individually separated, to our surprise, many ZGs from Cby1-KO mice were tethered by a proteinaceous material (arrowheads). Taken together, these results suggest that ablation of Cby1 causes secretory defects and ZG interlinkages, leading to cytoplasmic ZG accumulation and rapid acinar cell death.

## Discussion

We demonstrated that Cby1-KO mice exhibit rapid and progressive exocrine pancreatic degeneration, phenocopying the pancreatic lesions caused by ciliary defects in mouse models with a hypomorphic mutation of IFT88^30,32^ and a conditional deletion of KIF3A in the pancreas^31^. Shortly after birth, Cby1-KO pancreata show substantial exocrine degeneration, which progressively worsens into adulthood, leading to pancreatic atrophy with significant lipomatosis (Fig. 1). This coincides with inflammation, fibrosis, and ductal hyperplasia (Figs. 1, 2). We found that Cby1 localizes to the base of primary cilia (Fig. 5), and both number and length of primary cilia are significantly reduced in the pancreas of Cby1-KO mice (Fig. 6, Supplementary Fig. 3). Moreover, we provide evidence that the progressive loss of acinar cells may be attributable to defective secretion and accumulation of ZGs (Fig. 8). Our data are consistent with previous reports demonstrating that loss of cilia is associated with degeneration of acinar cells in the pancreas^30–32^.

What is the underlying mechanism of exocrine pancreatic degeneration in Cby1-KO mice? Cby1 localizes to the base of primary cilia and plays crucial roles in ciliogenesis in ductal and islet cells. Consistent with this, we found that, in the pancreas of Cby1-KO mice, Hh signaling is down-regulated while canonical Wnt signaling is up-regulated (Fig. 7). The elevated Wnt signaling response in the absence of Cby may be, in part, attributable to Cby1 function acting as an antagonist of β-catenin^14,17^. Interestingly, nonciliated acinar cells are most severely affected. The mechanistic connections between primary cilia and acinar cell death remain elusive. However, it was proposed that primary cilia in pancreatic ducts serve as mechanosensors to detect luminal flow, and impaired ciliary function could trigger ductal obstruction and dilation, leading to acinar cell death^30,31^.

Alternatively, a growing body of evidence suggests that ciliary proteins play cilia-independent roles in nonciliated cells^51,52^, and Cby1 may function in a cilium-independent manner in acinar cells. Indeed, we demonstrated that Cby1 is expressed in acinar cells (Supplementary Fig. 2). In response to secretory stimuli, acinar cells undergo a specialized form of exocytosis termed “sequential compound exocytosis”^50,53,54^. In this model, primary ZGs fuse with the luminal plasma membrane (primary exocytosis), followed by sequential fusion of secondary and tertiary ZGs with primary ZGs (compound exocytosis). The interconnection of ZGs is thought to yield a more rapid release of contents to the limited apical surface rather than discharge of individual ZGs. One possible interpretation of the interlinked ZGs purified from Cby1-KO acinar cells is the failure of ZG–ZG membrane fusion during compound exocytosis, resulting in defective ZG secretion (Fig. 8C). While Cby1-KO ZGs are interlinked via proteinaceous material, the ZG membranes do not appear to be fused (Fig. 8C, KO high mag.). This is in agreement with our model for the role of Cby1 in ciliogenesis in which Cby1 is involved in the efficient fusion of small vesicles to assemble a larger ciliary vesicle at basal bodies for basal body docking^5^. Although the impaired secretion and accumulation of ZGs in Cby1-KO mice might be caused secondarily by ciliary defects, it is also possible that loss of Cby1 may elicit direct effects on acinar cells in a cell-autonomous manner. Further experiments with acinar cell-specific deletion of Cby1 are necessary to distinguish between these possibilities.

The dysplasia, cysts, and fibrosis of the kidney, liver, and pancreas have been reported in ciliopathy patients^31,33,34^. About 10% of patients with autosomal dominant polycystic disease (ADPKD) exhibit pancreatic cysts^31,33,34,55^. Notably, it was also reported that approximately 70% of patients with von Hippel-Lindau (VHL) disease, an atypical ciliopathy and neoplastic syndrome, develop pancreatic cysts of varying numbers and sizes^34,35^. The Cby1-KO mouse model, therefore, provides a viable model to study the pancreatic condition of human ciliopathy patients.

## Methods

### Mouse strains and ethics statement

The Cby1-KO mouse line was created by replacing the entire coding region with a neomycin cassette as described previously^16^. Mice on a mixed C57BL/6J-129/SvJ background and FVB/NJ background were used since pancreatic exocrine degeneration was evident to a similar extent for both backgrounds. Mice were maintained on a 12-h light /12-h dark cycle in a specific pathogen-free facility, with ad libitum access to water and food. Age-matched littermates were used for all experiments. All mice were handled according to NIH guidelines, and all experimental procedures were approved by the Institutional Animal Care and Use Committee (IACUC) of the Stony Brook University and the University of Washington and in compliance with the ARRIVE guidelines.

### Histological analysis and immunohistochemistry

Mice were euthanized by CO_2_ asphyxiation, and the pancreas was dissected and fixed in 10% neutral buffered formalin or 4% paraformaldehyde (PFA). Samples were then processed for paraffin embedding. Five-μm sections were cut and stained with hematoxylin and eosin (H&E) and Masson’s trichrome.

Immunohistochemical staining of pancreatic paraffin sections was performed on a Ventana XT autostainer (Tucson), followed by counterstaining with hematoxylin. For IF staining, the pancreas was harvested, fixed in 4% PFA, and embedded in paraffin. Alternatively, the tissue was embedded in the Cryo-Gel medium (Instrumendics) and snap-frozen in a 2-methylbutaine bath in liquid nitrogen. Frozen sections were post-fixed with methanol-acetone (1:1). Sections were subjected to IF staining as described previously^16,17^. The following primary antibodies were used: CD45 (Millipore, 1:500), F4/80 (AbD Serotec, 1:500), cleaved caspase-3 (Cell Signaling, 1:500), Cby1 8–2 (in-house, 1:100)^56^, acetylated α-tubulin (Sigma-Aldrich, T7451, 1:1000), γ-tubulin (Sigma, T6557, 1:500), E-cadherin (BD Transduction Laboratories, 610182, 1:500), amylase (Sigma-Aldrich, A-8273, 1:500), insulin C-peptide (Millipore, 4023-01, 1:200), glucagon (Millipore, 4031-01F, 1:200), Arl13b (Proteintech, 17711-1-AP, 1:1000), and phospho-histone H3 (Sigma-Aldrich, 06-570, 1:2000). Alexa-conjugated secondary antibodies were purchased from ThermoFisher Scientific and used at a 1:500 dilution. Rhodamine-Peanut agglutinin (PNA) and fluorescein-*Dolichos biflorus* agglutinin (DBA) were purchased from Vector Laboratories and used at a 1:500 dilution. Cy5-DBA was purchased from GlycoMatrix and used at a 1:500 dilution. TUNEL assays were performed using a Click-iT TUNEL Alexa Fluor 594 Imaging Assay kit (ThermoFisher Scientific), according to the manufacturer’s instructions. Images were acquired using a Zeiss LSM510 or a Leica SP8X confocal microscope.

### Quantification of ciliary lengths

Pancreatic paraffin sections from P18 mice were labeled for A-tub and G-tub. Images were acquired with a 63 × objective using a DMI6000B epifluorescence microscope (Leica). Measurement of individual cilia was performed using the segmented line selection tool in ImageJ. A total of 51 cilia were quantified for ducts and islets for each genotype.

### BrdU incorporation assay

To determine proliferation in the pancreas, mice were given an intraperitoneal injection of 150 mg/kg BrdU (Sigma-Aldrich) and then euthanized 1 h later. Pancreatic frozen sections were post-fixed with methanol-acetone (1:1), treated with 2 N HCl for 30 min at room temperature, and processed for immunofluorescence staining with rat anti-BrdU antibody (Accurate, 1:300).

### Preparation of acini and exocytosis imaging using FM1-43

Isolation of dispersed pancreatic acini was performed by the enzymatic and mechanical dissociation technique using collagenase P (Roche) as described previously^57^. Isolated acini were seeded in Waymouth’s media (Sigma-Aldrich) supplemented with 0.1% BSA and 0.2 mg/ml soybean trypsin inhibitor (Sigma-Aldrich) in glass bottom dishes (MatTek Corporation) coated with Cell-Tak tissue cell adhesive (BD Biosciences). The acinar cells were then incubated with 2 μmol/l FM1-43 (Invitrogen) at 37 °C and imaged on a DMI6000B microscope (Leica) as described^58^. After obtaining stable basal fluorescence signals, cerulean (Sigma-Aldrich) was added to a final concentration of 1 nM to stimulate exocytosis of ZGs. Images were acquired every 1 min for 60 min.

### Isolation of zymogen granules (ZGs) and transmission electron microscopy (TEM)

ZGs were isolated from mouse pancreata as described^59^. The following buffer was used for homogenization: 250 mM sucrose, 5 mM MOPS, pH 7.0, 0.1 mM MgSO_4_, and 0.1 mM phenylmethylsulfonyl fluoride (PMSF), supplemented with protease inhibitor cocktail (Sigma-Aldrich). The tissue was then homogenized using a handheld tissue tearer. The homogenate was centrifuged at 500×*g* for 5 min at 4 °C, and the resulting post nuclear supernatant was further centrifuged at 2000×*g* for 15 min at 4 °C to sediment ZGs. The brownish layer of mitochondria on top of the ZG pellet was removed. The purified ZGs were fixed with 2% PFA and 2% glutaraldehyde in PBS, pH 7.4 and processed for TEM. TEM was conducted in the Central Microscopy Imaging Center at the Stony Brook University. Purified ZGs were fixed with 2% PFA and 2% glutaraldehyde in PBS, pH 7.4 and post-fixed in 2% osmium tetroxide, dehydrated, and embedded in Durcupan resin. Ultrathin sections of 80 nm were cut with a Reichert-Jung Ultracut E ultramicrotome and placed on formvar-coated slot copper grids. Sections were then counterstained with uranyl acetate and lead citrate and analyzed by a FEI Tecnai12 BioTwinG^2^ electron microscope. Digital images were acquired with an AMT XR-60 CCD Digital Camera System.

### Glucose measurements

Blood was collected from the tail vein and glucose concentration was measured using the FreeStyle Flash blood glucose monitoring system (Abbot Laboratories).

### Quantitative real-time PCR and RT-PCR

Total pancreatic RNA was isolated using RNeasy Mini Kit according to manufacturer’s instructions (Qiagen). Single-stranded cDNA was synthesized using the High Capacity cDNA Reverse Transcription Kit (Applied Biosystems). Real-time PCR was performed using the Fast SYBR Green Master Mix (Applied Biosystems) on a StepOnePlus Real-Time PCR System (Applied Biosystems). PCR primers used were as follows: Gli1, 5′-TTCACGCCTTGAAAACCTCAA-3′ and 5′-CAACCTTCTTGCTCACACATGTAAG-3′; Ptch1, 5′-CCTGCAAACCATGTTCCAGTT-3′ and 5′-TCGTAGCCCCTGAAGTGTTCA-3′; Axin2, 5′-CTCCCCACCTTGAATGAAGA-3′ and 5′-ACATAGCCGGAACCTACGTG-3′; and GAPDH, 5′-TCAACAGCAACTCCCACTCTTCCA-3′ and 5′-ACCCTGTTGCTGTAGCCGTATTCA-3′.

For RT-PCR, RNA was isolated from FACS-sorted pancreatic exocrine cells as described previously^60^. PCR primers used were as follows: Cby1, 5′-TCGACTATGGAACTCCTACC-3′ and 5′-CAGCAGAATGTCCACTTTCA-3′; and 18S ribosomal RNA (r18S), 5′-CTCAACACGGGAAACCTCAC-3′ and 5′-CGCTCCACCAACTAAGAACG-3′.

### Statistical analysis

Two-tailed Student’s t test was used for data analysis. In the figures, asterisks indicate p-values as follows: *p < 0.05; **p < 0.01; ***p < 0.001; and ****p < 0.0001.

## Supplementary Information

Supplementary Legends.

Supplementary Figure S1.

Supplementary Figure S2.

Supplementary Figure S3.

Supplementary Video 1.

Supplementary Video 2.
