# Supplementary material for: Loss of the ciliary protein Chibby1 in mice leads to exocrine pancreatic degeneration and pancreatitis
Source: Sci Rep. 2021 Aug 26;11:17220. doi: 10.1038/s41598-021-96597-w (PMC8390639; doi:10.1038/s41598-021-96597-w)
Supplement: Supplementary file 1 — Supplementary Legends. [file 41598_2021_96597_MOESM1_ESM.docx]

**Fig S1. Increased apoptosis and proliferation in the Cby1-KO pancreas.** **(A)** Pancreatic sections from P8 mice were subjected to TUNEL assays to evaluate the level of apoptosis (red). Nuclei were visualized with DAPI. Quantification represents the average of 10 fields (63x objective). Scale bar, 50 μm. **(B)** Pancreatic sections from adult mice were labeled with anti-phospho-histone H3 antibody (green) to examine the level of proliferation. The number of cells was counted in 10 fields (63x objective), and the percentage of phospho-histone H3-positive cells was calculated. Scale bar, 50 μm. Data represent means ± SEM. ** P < 0.01; *** P < 0.001.

**Fig S2. Cby1 expression in acinar cells.** **(A)** Expression levels of Cby1 and 18S ribosomal RNA (r18S, control) were assessed by RT-PCR analysis on RNA prepared from isolated acinar cells. PCR and agarose gel electrophoresis were performed at the same time, and the gel images were cropped. RT, reverse transcriptase. **(B)** The datasets for Cby1 expression in mouse acinar cells were retrieved from Wollny *et al* ^48^. The Y axis represents normalized Cby1 expression values, and the X axis indicates each acinar cell (a total of 113 acinar cells).

**Fig S3. Adult Cby1-KO pancreata exhibit a significant decrease in the number of primary cilia.** Pancreatic sections from adult mice were labeled for Arl13b (green) and A-tub (red) to assess the status of primary cilia in ducts and islets. Nuclei were visualized with DAPI. Scale bars, 20 μm and 10 μm (inset).

**Supplementary Videos S1 and S2. Defective ZG secretion in isolated Cby1-KO acini.** Isolated pancreatic acinar cells were stimulated with cerulean, subjected to live-cell imaging in the presence of the membrane fluorescent dye FM1-43. Arrows point to the acinar lumen.
