# Supplementary figures and images for: Loss of the ciliary protein Chibby1 in mice leads to exocrine pancreatic degeneration and pancreatitis
Source: Sci Rep. 2021 Aug 26;11:17220. doi: 10.1038/s41598-021-96597-w (PMC8390639; doi:10.1038/s41598-021-96597-w)

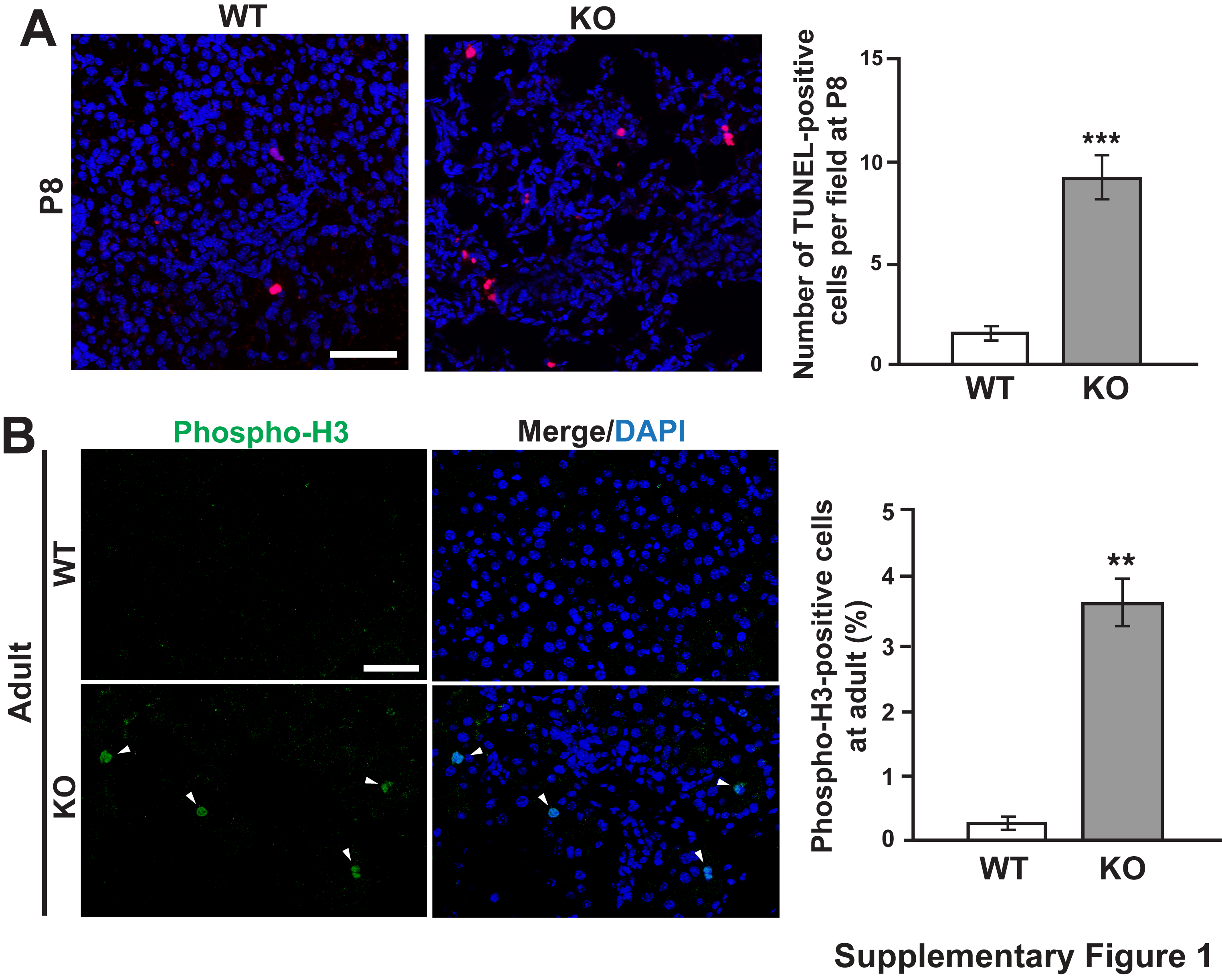

Supplement: Supplementary file 2 — Supplementary Figure S1. [file 41598_2021_96597_MOESM2_ESM.tif]

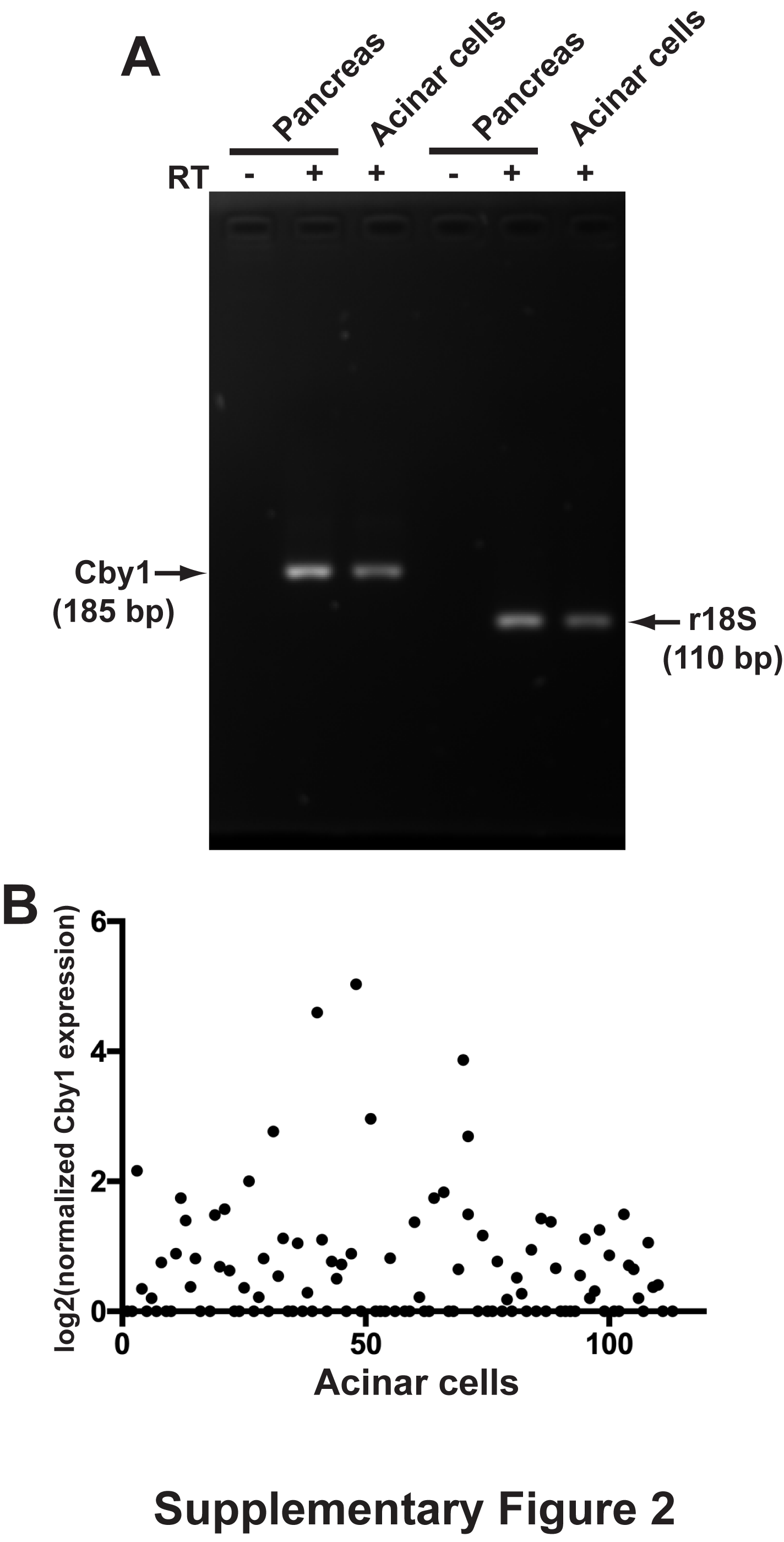

Supplement: Supplementary file 3 — Supplementary Figure S2. [file 41598_2021_96597_MOESM3_ESM.tif]

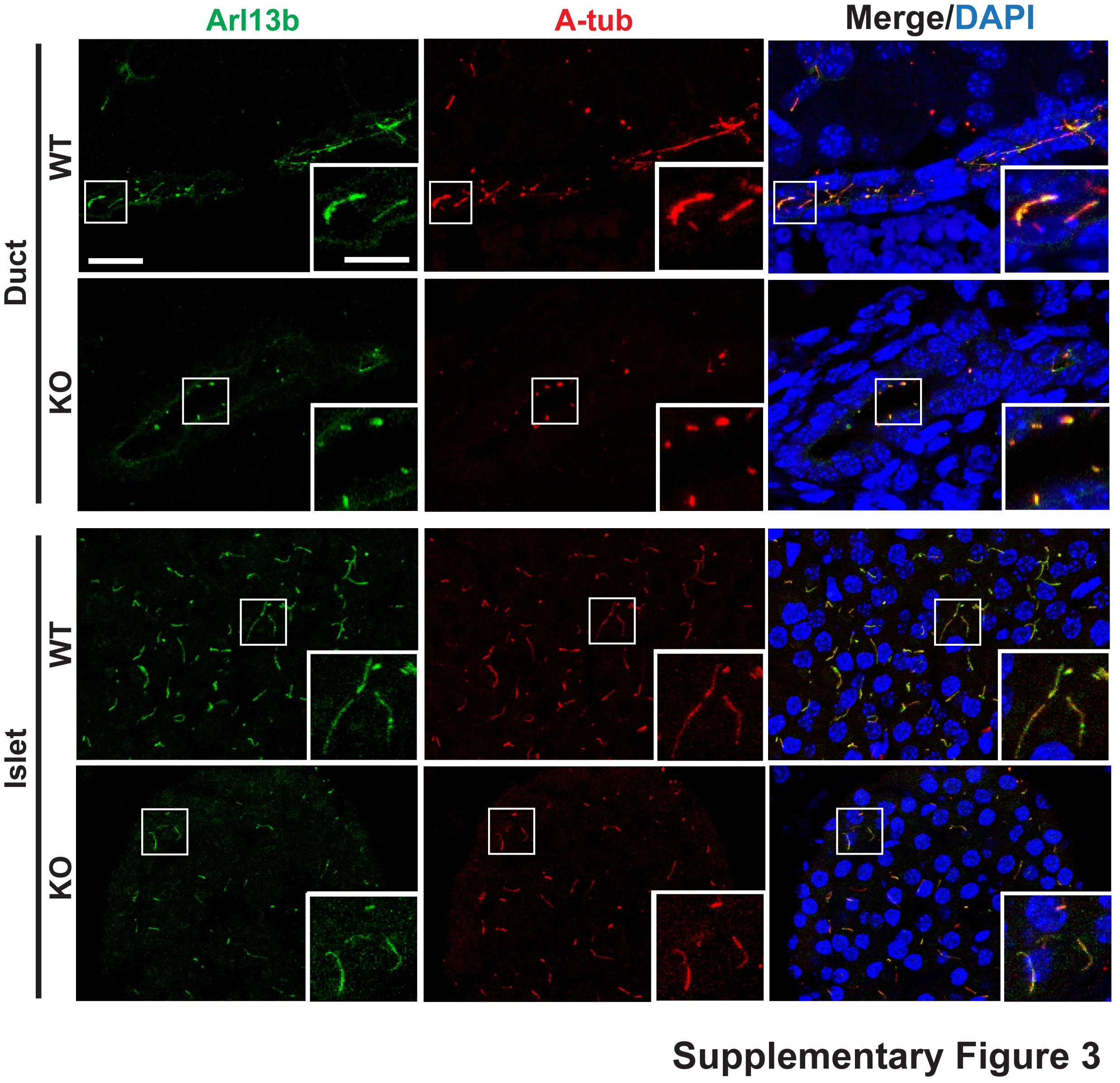

Supplement: Supplementary file 4 — Supplementary Figure S3. [file 41598_2021_96597_MOESM4_ESM.tif]
